# Structural Analysis and Inhibitor Modeling of Bacterioferritin From *Brucella abortus*

**DOI:** 10.1002/prot.70109

**Published:** 2026-01-02

**Authors:** Lijun Liu, Elizabeth K. Harmon, Justin K. Craig, Huili Yao, Kevin P. Battaile, David K. Johnson, Sandhya Subramanian, Wesley C. Van Voorhis, Mario Rivera, Scott Lovell

**Affiliations:** ^1^ Protein Structure and X‐Ray Crystallography Laboratory, Del Shankel Structural Biology Center University of Kansas Kansas USA; ^2^ Seattle Structural Genomics Center for Infectious Diseases Washington USA; ^3^ Center for Emerging and re‐Emerging Infectious Diseases (CERID), division of Allergy and Infectious Diseases, Department of Medicine University of Washington Washington USA; ^4^ Department of Chemistry Louisiana State University Louisiana USA; ^5^ New York Structural Biology Center New York New York USA; ^6^ Molecular Graphics and Modeling Laboratory and the Computational Chemical Biology Core University of Kansas Kansas USA

**Keywords:** bacterioferritin, brucellosis, inhibitor binding, iron transport, pathogens, protein complex

## Abstract

Iron homeostasis in various pathogenic bacteria is regulated by bacterioferritins (Bfr) which function to store Fe^3+^ and release Fe^2+^ as needed for metabolic processes. The Bfr structure consists of 18 kDa subunits in which dimer pairs bind a heme molecule and are assembled into a highly symmetrical 24‐meric spherical structure with an internal core diameter of approximately 80 Å. Release of iron is facilitated by the binding of a 7 kDa [2Fe‐2S] ferredoxin (Bfd) to specific sites on the surface of Bfr which transfers electrons to the core thereby reducing the stored Fe^3+^ to Fe^2+^ for mobilization. The crystal structures of Bfr from *Brucella abortus* (*Ba*) in the apo and iron bound forms are presented and compared with those from *Acinetobacter baumannii* (*Ab*) and *Pseudomonas aeruginosa* (*Pa*). Additionally, models of the Bfr:Bfd complexes for *Ba* and *Ab* are provided and compared with the *Pa* complex. Finally, compounds known to target the Bfr:Bfd interaction in *Pa* were docked to the *Ba* and *Ab* structures which provided insight regarding the potential binding mode and inhibitory mechanism.

**Abbreviations**
*Ab*
:
*Acinetobacter baumannii*

*Ba*
:
*Brucella abortus*

Bfd: bacterioferritin associated ferredoxin
Bfr: bacterioferritin
*Pa*
:
*Pseudomonas aeruginosa*

## 1 Introduction

The iron in the cytosol of bacteria is thought to contribute to three different pools, the free iron pool, the iron incorporated into cofactors and proteins, and the iron compartmentalized in iron storage proteins. The compartmentalization of iron in iron storage proteins is unique to iron homeostasis and is probably related to the reactivity of the soluble Fe^2+^ toward O_2_ and H_2_O_2_, and the extreme insolubility of Fe^3+^. Iron storage is conducted by ferritin‐like molecules which function by capturing Fe^2+^, oxidizing it to Fe^3+^, and internalizing it into their core cavities as a phosphate mineral. Hence, iron storage proteins can defend against iron‐induced toxicity while allowing the accumulation of iron at levels much higher than those allowed by the solubility of Fe^3+^ [1, 2]. Indeed, knock‐out and mutation studies have shown that iron storage proteins are essential for pathogenesis in vitro and in vivo for *Mycobacterium tuberculosis* [3]. The name ferritin describes a superfamily of proteins, as well as the specific iron storage protein in animals [2]. Despite low amino acid sequence conservation, the ferritins and the ferritin‐like molecules in bacteria share a conserved quaternary structure that results from the assembly of 24 subunits into spherical molecules that contain an ~80 Å diameter inner core, where more than 2000 Fe^3+^ ions can be stored. The genomes of most bacteria harbor genes encoding two types of ferritin‐like molecules, the bacterial ferritins (Ftn) and the bacterioferritins (Bfr). Bfr, present only in bacteria, consists of ~18 kDa alpha helical subunits that dimerize and bind a heme molecule by coordination with Met 52 of each subunit to the heme iron atom. The heme containing dimers further assemble into the biologically functional 24‐mer (440 kDa) sphere‐like structure with a hollow core where Fe^3+^ is compartmentalized [1, 4].

Although Bfr and Ftn were thought to coexist in bacteria as homopolymers, recent evidence has shown that the bacterioferritins from *Pseudomonas aeruginosa* and *Acinetobacter baumannii* are heteropolymers assembled from Ftn and Bfr subunits. *P. aeruginosa* Bfr (*Pa*‐Bfr) is a 24‐mer heteropolymer assembled from two types of subunit homodimers, FtnA and the heme binding BfrB (referred to as Bfr from this point forward) [5]. The crystal structure of *A. baumannii* Bfr (*Ab*‐Bfr), which is the first structural example of a heteropolymeric ferritin or ferritin‐like molecule, showed that the 24‐mer *Ab*‐Bfr is assembled from completely overlapping Ftn homodimers and heme‐containing Bfr homodimers devoid of ferroxidase centers [6]. The mobilization of Fe^3+^ compartmentalized in Bfr requires binding of Bfr‐associated ferredoxin (*Pa*‐Bfd) [7] at a site formed at the interface of each *Pa*‐Bfr subunit dimer, so that the [2Fe‐2S] cluster of *Pa*‐Bfd is placed in close proximity to the heme‐iron in each *Pa*‐Bfr subunit dimer [8].

Deletion of the *bfd* gene from the *P. aeruginosa* chromosome results in an irreversible accumulation of iron in *Pa*‐Bfr which is accompanied by intracellular iron depletion [9], an inability to form mature biofilms [10], and metabolic dysregulation [11]. These findings, together with the structure of the *Pa* Bfr‐Bfd complex [8], which revealed a conserved protein–protein interface (PPI) poised for disruption by small molecules, prompted us to implement a structure‐guided fragment‐based approach to discover PPI inhibitors [12]. This approach led us to 4‐aminoisoindoline‐1,3‐dione derivatives that can penetrate the bacterial cell, engage Bfr, dysregulate iron homeostasis, and kill *A. baumannii* and biofilm‐embedded *P. aeruginosa* cells [12, 13, 14].

In this study, the focus is on Bfr and Bfd from *Brucella abortus* which, along with other *Brucella* spp., cause Brucellosis in humans. Current treatments require prolonged therapy (6 weeks) with at least 2 antibiotics and have moderately high failure and relapse rates [15, 16]. Therefore, alternative targets for drug therapy are clearly needed for *Brucella* spp.

Given the high structural similarity amongst Bfrs and the conservation of residues at the Bfd binding site on Bfr observed in the Bfr and Bfd sequences of many pathogens [4, 13], the structural characterization of Bfr and inhibitor binding in Bfrs from other pathogens is expected to be useful for the development of currently available inhibitors into broad‐spectrum antibiotics. In this context, inspection of the *Brucella abortus* genome (strain 2308) revealed the presence of adjacent *bfr* and *bfd* genes at loci BAB2_0675 and BAB2_0676, respectively, which suggests functional attributes similar to *Pa‐*Bfr and *Pa*‐Bfd. It is therefore reasonable to hypothesize that inhibitors designed to bind at the Bfd binding site of *Pa*‐Bfr will also bind to the Bfd‐binding site of *Brucella abortus* Bfr (*Ba‐*Bfr) and disrupt iron homeostasis in *Brucella*. As a first step in this direction, in this study we report the X‐ray crystal structures of recombinant apo and iron bound *Brucella abortus* bacterioferritin (*Ba*‐Bfr) and discuss the similarities and differences relative to the *P. aeruginosa* (*Pa*‐Bfr) and *A. baumannii* (*Ab*‐Bfr) structures. We also present a model of the interactions between *Ba*‐Bfr and its bacterioferritin‐associated ferredoxin (*Ba*‐Bfd), which is similar to the *Pa* Bfr‐Bfd complex, suggesting that inhibitors designed to disrupt iron homeostasis in *P. aeruginosa* may exert similar effects in *Brucella abortus* .

## 2 Materials and Methods

### 2.1 Recombinant Protein Production and Purification

A construct coding for *Ba*‐Bfr (BrabA.00028.a.A1) spanning residues M1 to E160 (Uniprot Q2YKI4) was PCR‐amplified from gDNA and cloned into an AVA0421 vector which adds an N‐terminal hexahistidine purification tag and 3C protease cleavage site [17]. The plasmid DNA was transformed into chemically competent *Escherichia coli* BL21(DE3) Rosetta cells and expressed using 2 L of culture grown in auto‐induction medium [18] using a LEX Bioreactor (Epiphyte Three) as described previously [19]. It was discovered that an F16L mutation was inadvertently introduced during cloning, and this variant of the protein was used for all subsequent experiments.

*Ba‐*Bfr was purified using a previously described two‐step protocol consisting of an immobilized metal (Ni^2+^) affinity chromatography (IMAC) step followed by size‐exclusion chromatography (SEC) on an ÄKTApurifier 10 (GE Healthcare) using automated IMAC and SEC columns [19]. Bacterial pellets (25 g) were lysed by sonication in 200 mL of lysis buffer (25 mM HEPES pH 7.0, 500 mM NaCl, 5% (v/v) glycerol, 0.5% (w/v) CHAPS, 30 mM imidazole, 10 mM MgCl_2_, 400 μg/mL lysozyme, 3 U/mL benzonase). The crude lysate was treated with 20 mL (25 U/mL) of benzonase and incubated with mixing for 45 min at room temperature. The lysate was clarified by centrifugation at 12000 RCF for 1 h using a Sorvall centrifuge (Thermo Scientific) and loaded onto a HisTrap FF 5 mL column (GE Healthcare) equilibrated with 25 mM HEPES pH 7.0, 500 mM NaCl, 5% (v/v) glycerol, 30 mM imidazole. The column was washed with 20 column volumes (CV) of wash buffer and eluted with a linear gradient of 20 mM HEPES pH 7.0, 500 mM NaCl, 5% (v/v) glycerol, 500 mM imidazole over 7 column volumes. The fractions were incubated with 3C protease overnight to remove the His tag which leaves the extra residues GPGS upstream of the initiating methionine residue. The mixture was loaded onto a HisTrap FF column and the flow through containing the tagless protein pooled and concentrated to 5 mL.

The concentrated sample was loaded onto a Superdex 200 26/60 size exclusion column (GE Biosciences), equilibrated with 25 mM HEPES pH 7.0, 500 mM NaCl, 5% (v/v) glycerol, 2 mM DTT, 0.025% (w/v) sodium azide, using an ÄKTAprime plus FPLC system (GE Biosciences). The protein eluted from SEC in two symmetrical peaks with the first peak in the void volume and possibly due to the 24‐meric form. The second peak was consistent with the monomer. The peak fractions were collected and assessed for purity by SDS–PAGE, pooled and concentrated to 23 mg/mL. 110 μL aliquots of *Ba*‐Bfr were flash‐frozen in liquid nitrogen and stored at −80°C until ready for use.

### 2.2 Crystallization and Data Collection

Purified *Ba*‐Bfr, in 25 mM HEPES pH 7.0, 500 mM NaCl, 5% (v/v) glycerol, 0.025% (w/v) sodium azide was diluted to 10 mg/mL for crystallization screening. Excess hemin chloride (0.8 mM) was added to the protein and incubated for 1 h at room temperature prior to crystallization. All crystallization experiments were conducted using an NT8 drop‐setting robot (Formulatrix Inc.) and UVXPO MRC (Molecular Dimensions) sitting drop vapor diffusion plates at 17°C. 100 nL of protein and 100 nL crystallization solution were dispensed and equilibrated against 50 μL of the latter. Red prismatic crystals were obtained overnight from the following conditions and were cryoprotected as noted. ***Ba*‐Bfr‐Apo1**: Berkeley screen HT [20] condition H3: 20% (w/v) PEG 3350, 100 mM calcium chloride; cryoprotectant‐ 30% (w/v) PEG 3350 added to the crystallant; ***Ba*‐Bfr‐Apo2** and ***Ba*‐Bfr‐Fe2**: Berkeley screen HT condition G7: 1.5 M ammonium sulfate, 5% (v/v) MPD, 100 mM sodium acetate pH 4.5, cryoprotectant‐ 2.5 M lithium sulfate; ***Ba*‐Bfr‐Mg** and ***Ba*‐Bfr‐Fe1**: 21% (v/v) 2‐methyl‐2,4‐pentanediol, 400 mM MgCl2, 100 mM sodium acetate pH 4.6, cryoprotectant‐ 35% (v/v) MPD added to the crystallant. To prepare the iron complexes, crystals from the conditions noted above were soaked for 5 min in 12.5 mM ferrous ammonium sulfate in crystallant. X‐ray diffraction data were collected at the National Synchrotron Light Source II (NSLS‐II) beamline 19‐ID‐I (NYX) using a Dectris Eiger2 9 M XE pixel array detector.

### 2.3 Structure Solution and Refinement

Intensities were integrated using XDS [21, 22] via autoPROC [23] and the Laue class analysis and data scaling were performed with Aimless [24]. Structure solution was conducted by molecular replacement with Phaser [25] using a previously determined *Ba*‐Bfr structure (PDB 3FVB) as the search model. Structure refinement and manual model building were conducted with Phenix [26] and Coot [27] respectively. Structure validation was conducted with Molprobity [28] and figures were prepared using the CCP4MG package [29]. Structure superpositions were conducted using secondary structure matching using GESAMT [30, 31]. Crystallographic data are provided in Table 1.

**TABLE 1:** Crystallographic data for *Ba*‐Bfr structures.

Sample	*Ba*‐Bfr‐Apo1	*Ba*‐Bfr‐Apo2	*Ba*‐Bfr‐Mg	*Ba*‐Bfr‐Fe1	*Ba*‐Bfr‐Fe2
PDB code	8SQP	8SQQ	8SQO	8SQR	8SQT
Data collection					
Unit‐cell parameters (Å, ^o^)	*a* = *b* = *c* = 112.07 *α* = *β* = *γ* = 90	*a* = *b* = *c* = 171.52 *α* = *β* = *γ* = 90	*a* = *b* = *c* = 112.91 *α* = *β* = *γ* = 90	*a* = *b* = *c* = 113.03 *α* = *β* = *γ* = 90	*a* = *b* = *c* = 170.72 *α* = *β* = *γ* = 90
Space group	*P*432	*F*432	*P*432	*P*432	*F*432
Resolution (Å) [1]	45.75–2.05 (2.11–2.05)	42.88–2.25 (2.32–2.25)	46.10–1.55 (1.58–1.55)	46.15–1.65 (1.68–1.65)	49–28‐2.20 (2.27–2.20)
Wavelength (Å)	0.9795	0.9795	0.9795	0.9795	0.9795
Temperature (K)	100	100	100	100	100
Observed reflections	615 703	419 133	2 790 308	1 761 720	441 158
Unique reflections	15 708	10 780	36 301	30 324	11 356
<I/σ(I)> [1]	28.2 (1.5)	18.1 (1.9)	24.2 (1.8)	22.8 (1.7)	26.3 (1.9)
Completeness (%) [1]	100 (100)	100 (100)	100 (100)	100 (100)	100 (100)
Multiplicity [1]	39.2 (41.8)	38.9 (41.2)	76.9 (80.5)	58.1 (60.9)	38.8 (41.5)
*R*_merge_ (%) [1, 2]	10.1 (335.9)	20.6 (285.7)	18.5 (427.0)	16.7 (393.9)	14.2 (270.6)
*R*_meas_ (%) [1, 4]	10.2 (340.0)	20.9 (289.2)	18.7 (429.7)	16.8 (397.2)	14.3 (274.0)
*R*_pim_ (%) [1, 4]	1.6 (52.3)	3.3 (44.8)	2.1 (47.7)	2.2 (50.7)	2.3 (42.3)
CC_1/2_ [1, 5]	1.000 (0.661)	0.999 (0.668)	1.000 (0.731)	1.000 (0.728)	1.000 (0.693)
Refinement					
Resolution (Å) [1]	31.08–2.05	39.35–2.25	28.23–1.55	32.63–1.65	49.28–2.20
Reflections (working/test) [1]	14 882/812	10 253/520	34 522/1765	28 819/1488	10 792/553
*R*_factor_ / *R*_free_ (%) [1, 3]	19.6/21.5	18.9/24.6	16.1/17.7	17.3/18.6	19.4/26.2
No. of atoms (Protein/Fe^2+^ or Mg^2+^ /Water)	1277/−/30	1294/−/29	1331/2/142	1328/5/122	1291/3/29
Model quality					
R.m.s deviations					
Bond lengths (Å)	0.003	0.013	0.013	0.008	0.012
Bond angles (^o^)	0.551	1.139	1.241	0.902	1.070
Mean *B*‐factor (Å^2^)					
All Atoms	55.7	48.9	27.3	30.0	54.6
Protein	55.8	48.7	25.9	28.6	54.1
Fe^2+^ or Mg^2+^	—	—	28.9	58.2	113.9
Water	52.9	49.6	38.3	39.0	57.8
Coordinate error (maximum likelihood) (Å)	0.21	0.25	0.13	0.19	0.22
Ramachandran Plot					
Most favored (%)	100	100	98.1	100	100
Additionally allowed (%)	—	—	1.9	—	—

*Note*: Values in parenthesis are for the highest resolution shell. *R*_merge_ = ∑_*hkl*_∑_*i*_ |*I*_*i*_(*hkl*) ‐ < *I*(*hkl*) > | / ∑_*hkl*_∑_*i*_ *I*_*i*_(*hkl*), where *I*_*i*_(*hkl*) is the intensity measured for the *i*th reflection and < *I*(*hkl*) > is the average intensity of all reflections with indices hkl. *R*_factor_ = ∑_*hkl*_ ||*F*_obs_ (*hkl*) | ‐ |*F*_calc_ (*hkl*) || / ∑_*hkl*_ |*F*_obs_ (*hkl*)|; Rfree is calculated in an identical manner using 5% of randomly selected reflections that were not included in the refinement. *R*_meas_ = redundancy‐independent (multiplicity‐weighted) *R*_merge_ [24, 32]. *R*_pim_ = precision‐indicating (multiplicity‐weighted) *R*_merge_ [33, 34]. CC_1/2_ is the correlation coefficient of the mean intensities between two random half‐sets of data [35, 36].

### 2.4 Heme Reconstitution of *Ba*‐Bfr

Six μL of a hemin solution (2 mg/mL in DMSO (Alfa Aesar)) was added to a 0.5 mL, 5.7 μM solution of *Ba*‐Bfr in 100 mM potassium phosphate containing 1 mM TCEP (pH 7.5). The resulting mixture was gently mixed by inverting the Eppendorf tube a few times. The process was repeated two more times while monitoring the UV spectrum of the sample after each addition of hemin. The resultant mixture was centrifuged for 10 min at 16200 × *g*, followed by loading the supernatant onto a Superdex 200 Increase 10/300 GL size exclusion column equilibrated with 100 mM potassium phosphate containing 1 mM TCEP (pH 7.5). The protein eluting from the column exhibited a ratio of absorbances (A_280_/A_418_) ~0.9.

### 2.5 Ligand Docking

Apo protein structures of *Ba*‐Bfr (PDB 8SQP) and *Ab*‐Bfr (PDB 7K5H [6]) were aligned to the *Pa*‐Bfr structure in complex with inhibitor KM‐5‐35 (PDB 9NHT [14]) and KM‐5‐35 ligand was merged into each protein structure. Proteins were loaded into Maestro [37] and clashing protein residues near the superimposed ligands were minimized. The protein‐ligand complexes were then prepared using the protein preparation wizard. Docking grids were centered on the KM‐5‐35 ligand and both KM‐5‐35 and KM‐5‐66 were prepared using LigPrep [37]. The ligands were docked into each receptor using a core constraint composed of the phthalimide group interactions with the protein similar to those observed in the 9NHT structure. Docked poses were inspected visually and a top pose was selected for each protein‐ligand combination. Ligand binding affinities were predicted using Boltz‐2 [38].

## 3 Results

### 3.1 Bfr and Bfd Sequence Comparisons

**FIGURE 1:**
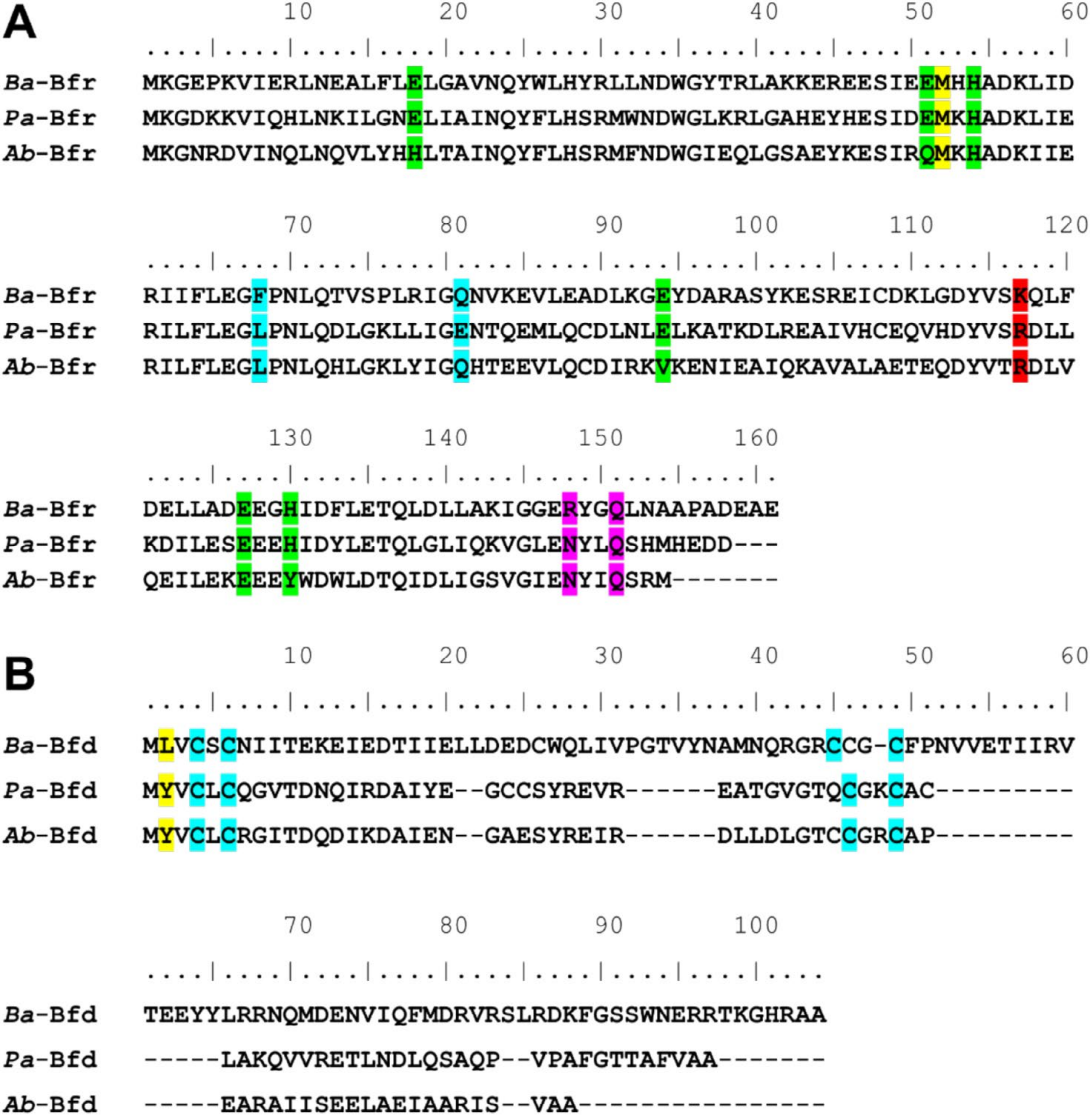
Bfr and Bfd sequence alignments. (A) Amino acid sequence alignment of *Ba*, *Pa* and *Ab* Bfr. Residues in the ferroxidase center are highlighted green, the residues that form the Bfd binding pocket are cyan, the 4‐fold pore residues in magenta and 3‐fold pore residues in red. The methionine residue that binds heme highlighted in yellow. (B) Amino acid sequence alignment of *Ba*, *Pa* and *Ab* Bfd. Cysteine residues that bind the 2Fe‐2S cluster are highlighted cyan and the residue that binds into the Bfr pocket is highlighted yellow.

Alignment of the *Ba‐*Bfr (Uniprot Q2YKI4) amino acid sequence with *Pa‐*Bfr (Uniprot Q9HY79) and *Ab‐*Bfr (Uniprot A0A1E3M9R5) revealed a 51% and 44% identity respectively (Figure 1A). Notably, the six residues that function as ligands of the ferroxidase di‐Fe center, which catalyzes the oxidation of Fe^2+^ to Fe^3+^, are completely conserved between *Ba‐*Bfr and *Pa‐*Bfr. However, only three of the six residues in the ferroxidase center are conserved for *Ab‐*Bfr, which may suggest a different iron binding mode or the absence of a ferroxidase center. The structure of *Ab*‐Bfr revealed that the protein is a heterooligomer composed of *Ab* ferritin homodimers and *Ab* bacterioferritin homodimers. The ferritin subunits harbor ferroxidase centers identical to *P. aeruginosa* ferritin (*Pa*‐Ftn), whereas the bacterioferritin subunits do not have a full complement of ferroxidase ligands and most likely do not possess a functional ferroxidase center. Hence, in *Ab‐*Bfr, the ferritin subunits function in Fe^2+^ oxidation and Fe^3+^ storage, while the Bfr subunit dimers and associated heme bind Bfd and enable Fe^3+^ reduction and Fe^2+^ mobilization to the cytosol [6].

Alignments of the *Ba‐*Bfd (Uniprot Q2YKI3) amino acid sequence with *Pa‐*Bfd (Uniprot Q9HY80) and *Ab‐*Bfd (Uniprot D0CCQ2) revealed an 18% and 20% identity respectively (Figure 1B). The *Ba‐*Bfd contains 103 amino acids whereas the *Pa‐*Bfd and *Ab‐*Bfd sequences have 73 and 69 residues respectively. Notably, this isoform of *Ab‐*Bfd contains five extra residues (MGITP) at the N‐terminus that are not present in the other Bfd proteins and the Bfd domain spans residues 6–69. Therefore, for the purpose of uniformity, residue M6 is considered residue M1 in our comparison. Although the Bfd sequences differ significantly, the cysteine residues that bind the [2Fe‐2S] cluster are conserved. Additionally, the amino acid that is known to occupy the Bfr binding site at position 2 is a Tyr residue in *Pa‐*Bfd and *Ab‐*Bfd but is a Leu in *Ba‐*Bfd.

### 3.2 *Ba*‐Bfr Structure

**FIGURE 2:**
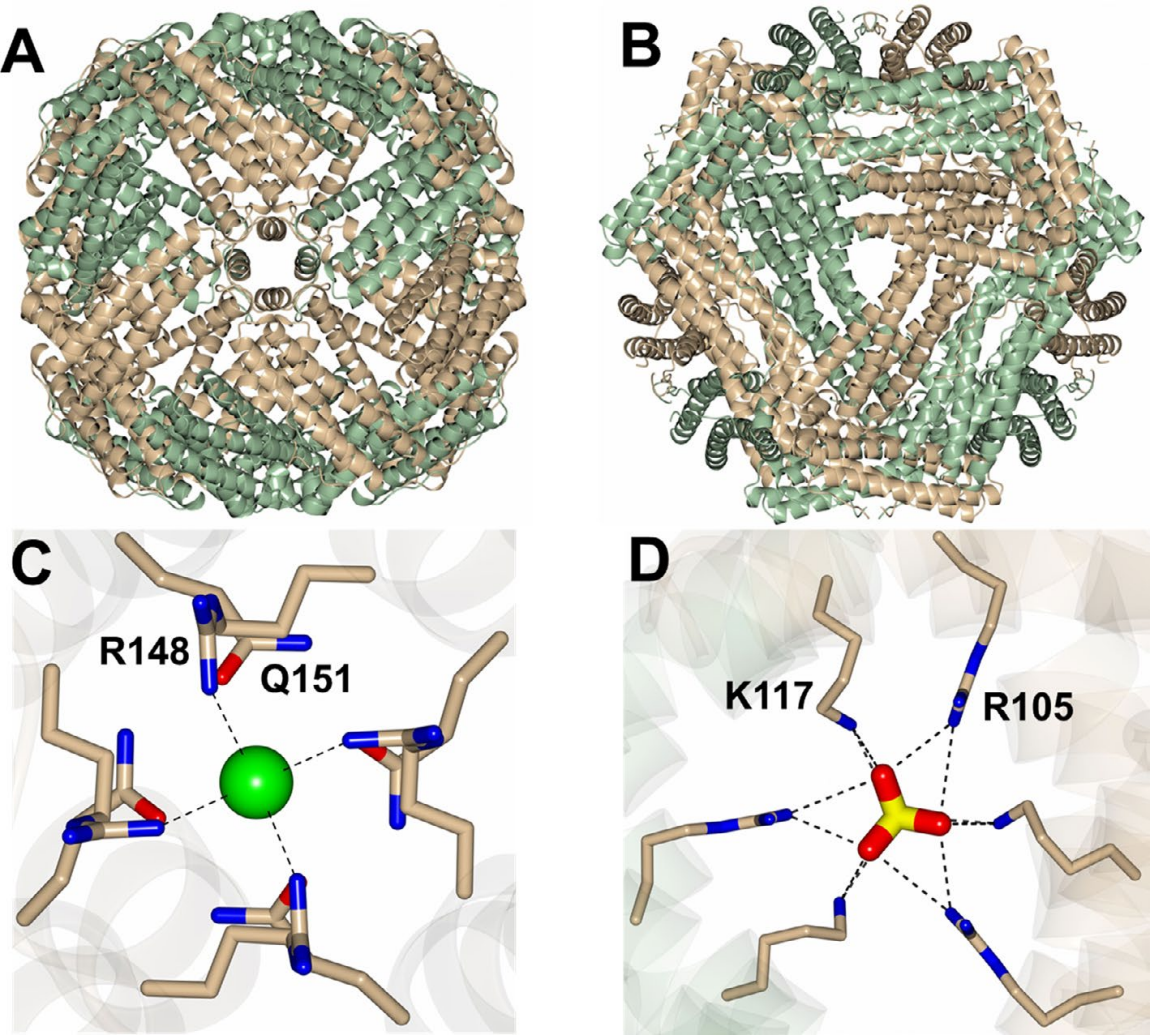
Structure of *Ba*‐Bfr showing the 24‐meric structure viewed along a (A) 4‐fold pore and (B) 3‐fold pore. Interactions between *Ba*‐Bfr and (C) a chloride and (D) sulfate ions observed in the 4‐fold and 3‐fold pores respectively.

The *Ba*‐Bfr structures described here include two apo (*Ba*‐Bfr‐Apo1 and *Ba*‐Bfr‐Apo2), a magnesium bound (*Ba*‐Bfr‐Mg), and two iron bound structures (*Ba*‐Bfr‐Fe1 and *Ba*‐Bfr‐Fe2) that crystallized in two different cubic lattices (Table 1). *Ba*‐Bfr‐Apo1, *Ba*‐Bfr‐Fe1, and *Ba*‐Bfr‐Mg belong to one crystal form (primitive cubic), and *Ba*‐Bfr‐Apo2 and *Ba*‐Bfr‐Fe2 to the other form (face‐centered cubic). It was noted in the experimental section that an F16L mutation was found in the clone used for crystallization. However, this form does not influence the Bfr structure as comparison with a WT structure determined in 2009 by our SSGCID group (SSGCID, 3FVB) yielded an RMS deviation of 0.27 Å (157 residues) between Cα atoms. All structures contained a monomer in the asymmetric unit, and the application of the space group symmetry operators produces the 24‐meric biological assembly (Figure 2A,B) containing 4‐fold and 3‐fold pores as observed for various Bfr structures. Interestingly, the purified *Ba*‐Bfr protein was mainly composed of dimers as determined by size exclusion chromatography but was not fully loaded with heme as determined by the A420 nm/A280 nm ratio (Figure S1A,B). However, titration with heme resulted in the formation of a small population of 24‐mers and an increase in heme occupancy (Figure S1C,D). The inability to drive the equilibrium to the formation of mainly 24‐mers is unknown. We speculate that heme incorporation into the dimer in vitro helps organize (compact) the dimer such that 24‐mer assembly is partially enabled. Therefore, the crystals obtained were likely generated from the small amount of 24‐mers in the mixture.

The various *Ba*‐Bfr structures were found to be structurally similar overall where the superposition of the structures onto *Ba*‐Bfr‐Apo1 yielded the following RMS deviations between Cα atoms: *Ba*‐Bfr‐Apo2; 0.22 Å (157 residues), *Ba*‐Bfr‐Mg; 0.18 Å (156 residues), *Ba*‐Bfr‐Fe1; 0.41 Å (156 residues), and *Ba*‐Bfr‐Fe2; 0.22 Å (157 residues). Notably, the 4‐fold pores, formed by R148 and Q151, contain a modeled chloride ion coordinated by the R148 residue (Figure 2C). The 4‐fold pores in *Pa‐*Bfr and *Ab‐*Bfr are lined by residues N148 and Q151, and the various *Pa‐*Bfr structures contain a potassium ion coordinated by these residues. The positively charged R148 residue may preferentially bind anions, although the specific ion type can be difficult to distinguish from an electron density map. However, the presence of arginine at this position seems to be unique to *Ba*‐Bfr as a Blast [39, 40] search against the PDB revealed that the majority of Bfr structures contain an asparagine residue at this site along with a few structures harboring a leucine or glycine (Figure S2). The 3‐fold pores in *Ba*‐Bfr, generated by R105 and K117, coordinate a sulfate ion (Figure 2B,D), which is similar to *Pa‐*Bfr and *Ab‐*Bfr that contain an R117 residue that coordinates a sulfate ion in a similar manner [41]. Notably, the *Ba*‐Bfr‐Apo1 structure did not contain sulfate in the crystallization solution but had high concentrations of calcium, which was modeled at the 3‐fold pore for this structure. However, it may be that a mixture of Ca^2+^ and Cl^−^ ions are present at this site with a preference for the latter.

**FIGURE 3:**
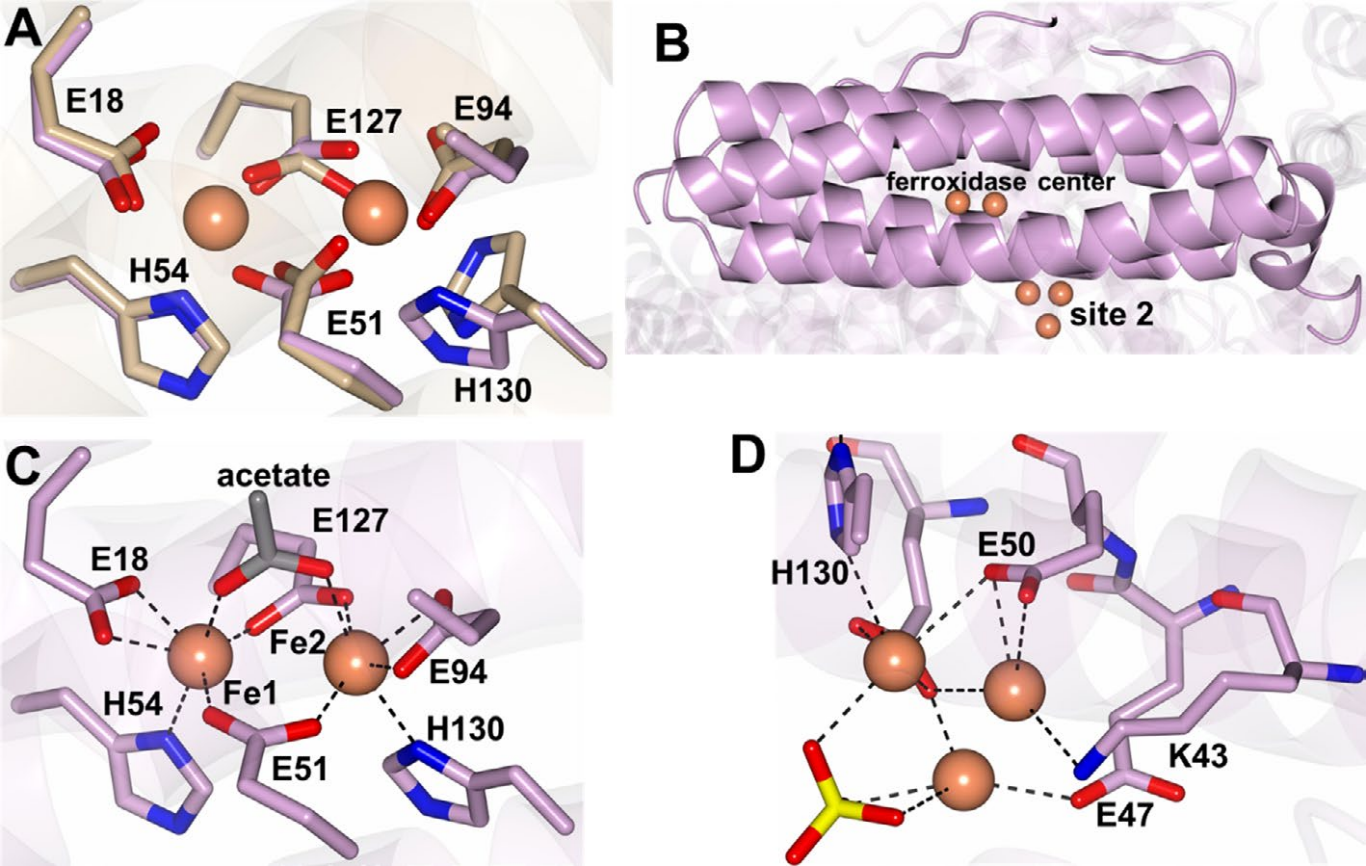
Iron binding in the *Ba*‐Bfr‐Fe1 structure. (A) Superposition showing conformational difference between the ferroxidase center ligands in *Ba*‐Bfr‐Apo1 (tan) and *Ba*‐Bfr‐Fe1 (plum). (B) Highlighted subunit showing the location of the site 2 Fe cluster relative to the ferroxidase center ions. The interactions with the Fe ions in the (C) ferroxidase center and (D) site 2 iron binding regions. The sulfate ion in site 2 is drawn as yellow/red cylinders.

The *Ba*‐Bfr crystals soaked in solutions containing iron produced large peaks of electron density in the ferroxidase center that also displayed an anomalous signal in a phased anomalous difference map (Figure S3A). Iron binding results in small conformational changes in the ferroxidase center side chains when comparing the *Ba*‐Bfr‐Apo1 and *Ba*‐Bfr‐Fe1 structures (Figure 3A). Additionally, a second site containing a cluster of iron ions was observed for *Ba*‐Bfr‐Fe1 which is located near the interior of the Bfr core (Figure 3B and Figure S3B). The iron ion Fe1 is coordinated by E18 and H54 and Fe2 is coordinated by E94 and H130. Residues E51 and E127 bridge both iron ions along with an acetate ion (Figure 3C). The iron ions in the interior site are coordinated by K43 E47, E50 and H130 (Figure 3D). A structure of *Pa*‐Bfr (PDB 4TOH) also contained an iron ion in a similar secondary site coordinated by H48 [41]. The *Ba*‐Bfr‐Fe2 structure displayed similar electron density for two iron ions in the ferroxidase center as well as the secondary site (Figure S4A). The main difference is a small conformational change in the E127 side chain, which is rotated relative to *Ba*‐Bfr‐Fe1 but still bridges the ferroxidase iron ions. Additionally, a bridging water molecule was modeled in this structure.

The *Ba*‐Bfr‐Mg structure displayed electron density near the ferroxidase center that was modeled as a Mg^2+^ ion which was acquired from the crystallization solution (Figure S4B). This density was not observed in *Ba*‐Bfr‐Apo1 or *Ba*‐Bfr‐Apo2 structures and therefore was most likely not coordinated during expression or purification. The Mg^2+^ ion is located near the ferroxidase center and forms contacts with H54, E127, and H130 as well as D126 which is outside of the ferroxidase center. Similar conformations in the ferroxidase center residues were observed when compared to the *Ba*‐Bfr‐Fe1 structure.

### 3.3 Comparison With *Pa* and *Ab* Bfr

**FIGURE 4:**
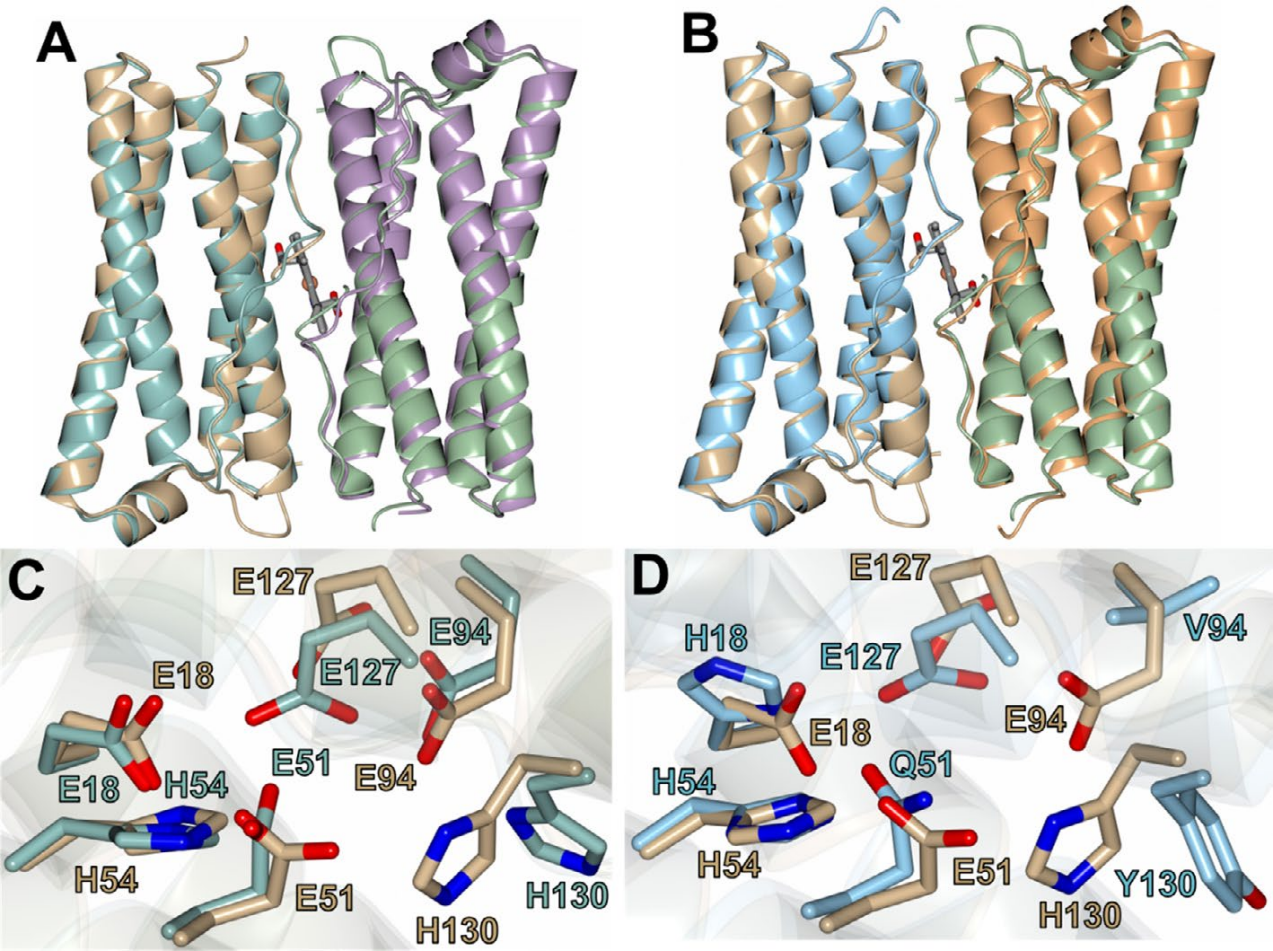
Comparison of the *Ba*‐Bfr structure (tan/green) with *Pa*‐Bfr (teal/purple, 3IS7) and *Ab*‐Bfr (blue/gold, 9BTS). (A) and (B) Superpositions showing the similarity to the *Pa*‐Bfr and *Ab*‐Bfr structures respectively. The heme molecules between the dimers are rendered as gray cylinders. (C) and (D). Comparison of the ferroxidase center protein ligands for *Pa*‐Bfr and *Ab*‐Bfr respectively.

The *Ba*‐Bfr structures were found to be structurally similar to the *Pa*‐Bfr (3IS7) and *Ab*‐Bfr (9BTS) structures with RMS deviations between Cα atoms of 0.67 Å (151 residues) and 0.78 Å (152 residues) for 3IS7 and 9BTS respectively. The dimer pairs are nearly identical in the α‐helical regions and only differ slightly in the flexible linker portions (Figure 4A,B). As mentioned previously, the six residues that comprise the ferroxidase center, which is the region where iron oxidation occurs, are completely conserved between *Ba‐*Bfr and *Pa‐*Bfr, and are composed of residues E18, E51, H54, E94, E127, and H130. However, in *Ab‐*Bfr these residues correspond to H18, Q51, H54, V94, E127, and Y130. The localization of the ferroxidase center residues is quite similar for all structures as shown in Figure 4C,D.

Apart from the coordination of the acetate ion observed in *Ba‐*Bfr, which was likely acquired from the crystallization solution, the iron coordination is nearly identical to the binding mode observed for *Pa‐*Bfr (PDB 3IS8) as shown in Figure S5. The main difference is observed at residue H130 which was shown to adopt “gate open and closed” conformations in *Pa‐*Bfr to facilitate iron mobilization to the core [42]. Conversely, *Ba*‐Bfr undergoes an approximately 90° rotation of the H130 side chain to coordinate the Fe2 iron ion and one of the iron ions in the secondary site (see Figure 3).

### 3.4 Comparison and Modeling of the *Ba*‐Bfr Interaction With Bfd

**FIGURE 5:**
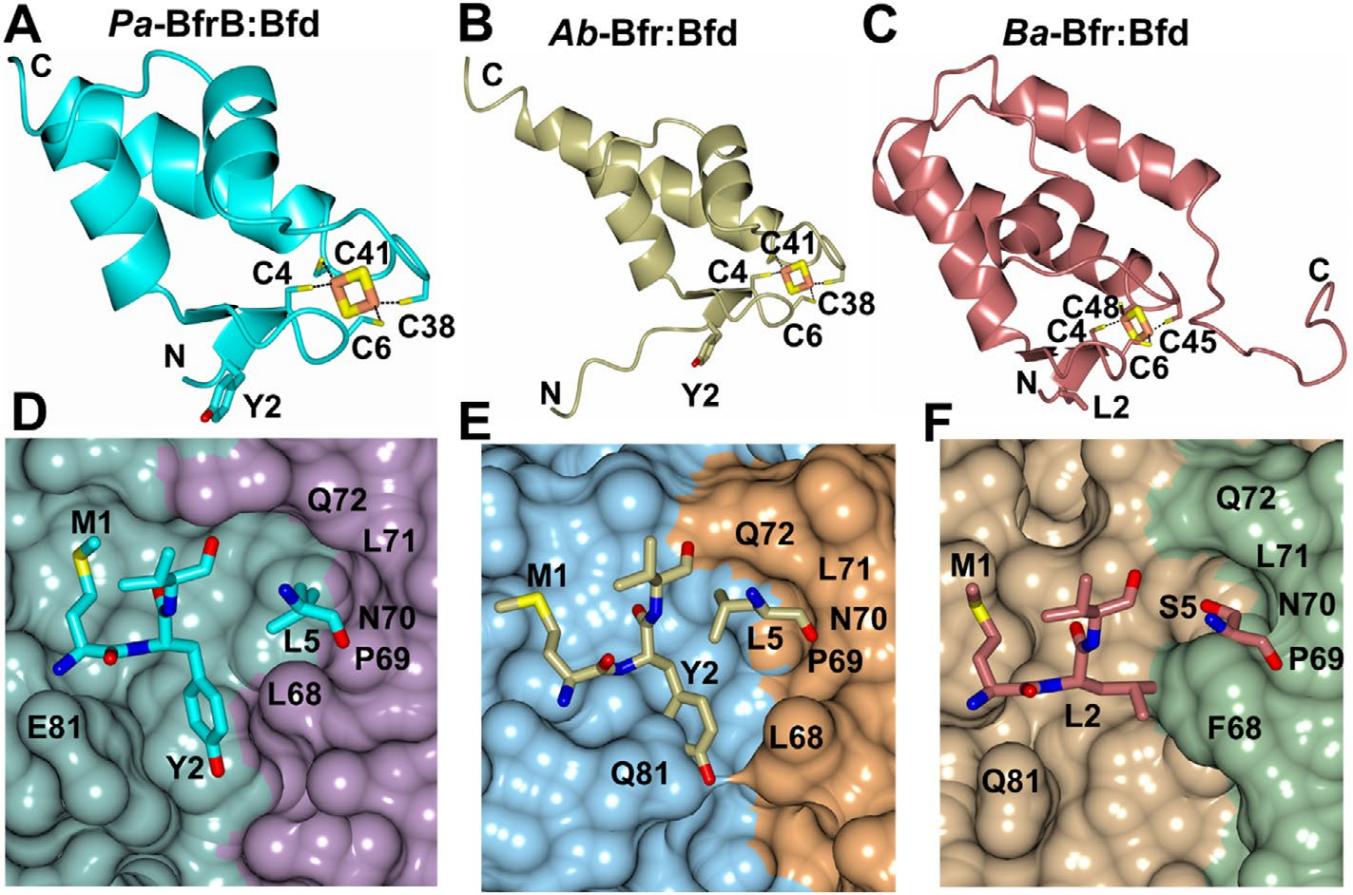
Comparison of the Bfd structures and interactions with Bfr. (A, D) *Pa*‐Bfr:Bfd, (B, E) *Ab*‐Bfr:Bfd and (C, F) *Ba*‐Bfr:Bfd. Panels A–C show the Bfd structures along with the Cys residues that interact with the 2Fe‐2S cluster (yellow/coral cylinders). Panels D–F show the locations of the Bfd residues (cyan, gold and red) in the cleft formed by residues 68 and 81 from Bfr dimer subunits. Residues P69‐Q72 in one if the Bfr subunits are located near the phthalimide binding pocket.

The *Pa‐*Bfr in complex with its bacterioferritin‐associated ferredoxin (Bfd) provided insight regarding the mechanism of electron transport to the core of Bfr to facilitate iron mobilization [43]. This structure (PDB 4E6K) is composed of a *Pa‐*Bfr 24‐mer with 12 Bfd subunits positioned above the heme molecules of the Bfr dimers. The [2Fe‐2S] cluster of Bfd is coordinated by four conserved cysteine residues that are also present in *Ab* and *Ba*‐Bfd. Importantly, residue Y2 from Bfd is positioned between residues L68 and E81 from neighboring Bfr subunits which forms part of the protein–protein interaction interface (PPI) as shown in Figure 5A,D. Additionally, it was shown that L5 from Bfd is one of the residues with the largest buried surface area and occupies a cleft between Bfr dimers near residues P69‐Q72. This site comprises the region where the phthalimide group of current inhibitors binds and forms critical hydrogen bond interactions with Bfr [1, 13, 14]. *Ab* (Uniprot D0CCQ2) and *Ba* (Uniprot Q2YKI3) Bfd models generated using Alphafold3 [44] were superimposed onto a Bfd subunit of the *Pa‐*Bfr:Bfd structure, which yielded RMS deviations of 0.49 Å (55 residues) and 1.31 Å (44 residues) between Cα atoms, respectively for *Ab* and *Ba* Bfd. Notably, the folds are very similar amongst the Bfd structures, with each containing three central α‐helices, although *Ba*‐Bfd has an extra C‐terminal helix which results from additional residues in this region relative to *Pa* and *Ab* Bfd (Figure 5A–C). Similar to the *Pa*‐Bfr:Bfd structure, the models constructed for the *Ab* and *Ba* Bfr:Bfd complexes would position residues Y2 and L2, respectively, in the Bfr PPI site formed by L68/Q81 (*Ab*) and F68/Q81 (*Ba*), as can be observed in Figures 5E,F. Moreover, the L5 and S5 residues of *Ab* and *Ba* Bfd, respectively, are positioned in the pocket where the phthalimide moiety of inhibitors bind *Pa*‐Bfr. This suggests that the *Ab* and *Ba* Bfr:Bfd complexes likely utilize a similar mechanism as *Pa* for iron mobilization which may be similarly affected by the binding of current *Pa* Bfr‐Bfd protein–protein interaction inhibitors.

### 3.5 Inhibitor Docking

**FIGURE 6:**
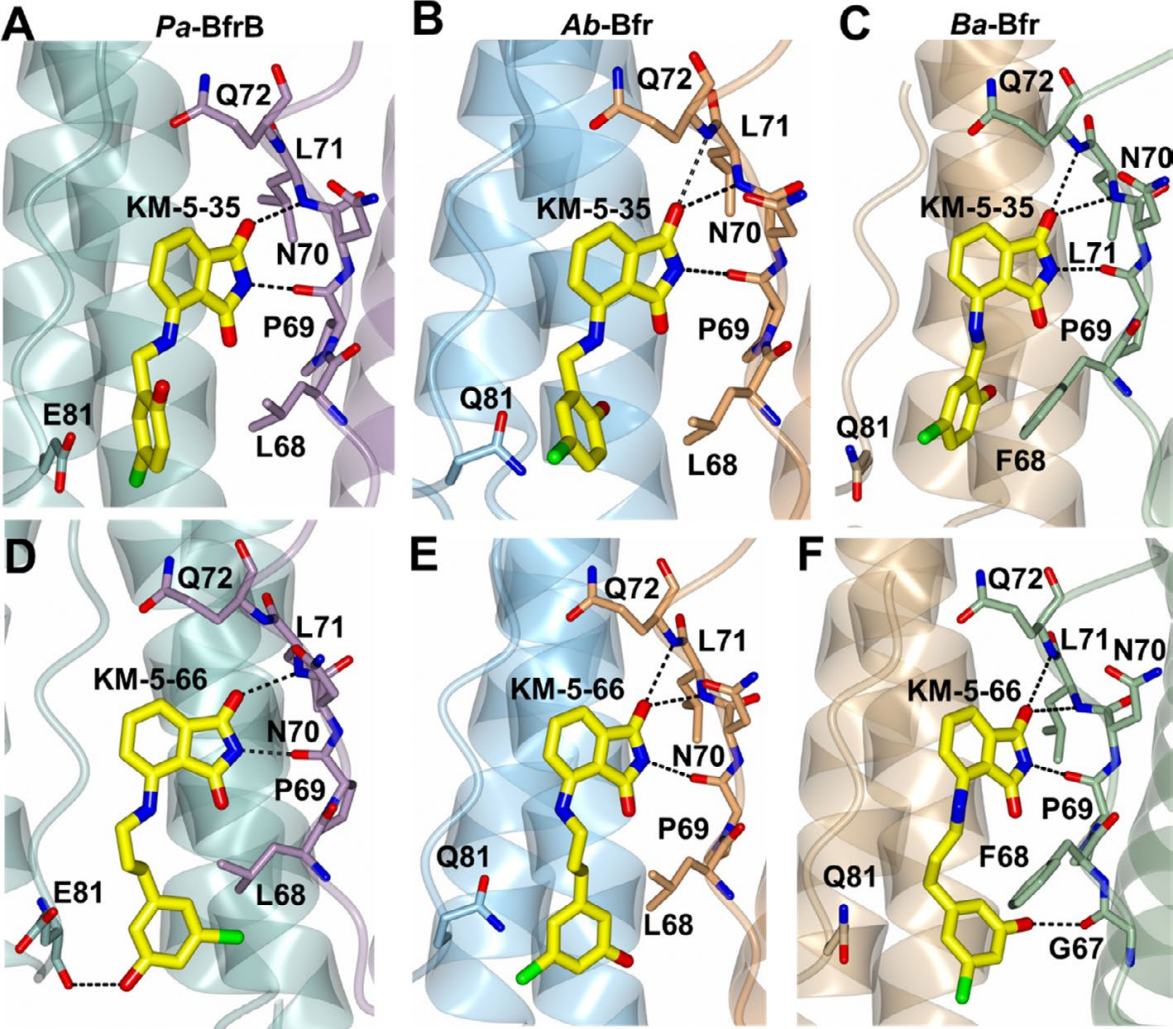
Ligand docking (yellow) of inhibitors KM‐5‐35 (A–C) and KM‐5‐66 (D–F). *Pa*‐Bfr, *Ab*‐Bfr and *Ba*‐Bfr are shown in panels (A, D), (B, E) and (C, F) respectively.

The *Pa*‐Bfr:Bfd structure provided a platform for the development of novel protein: protein interaction inhibitors based on the interacting surfaces which have been exploited using structure‐based drug design methods [1, 12, 13, 14]. The basis of these inhibitors is found in the phthalimide group which forms distinct hydrogen bond interactions with P69 and L71 located at the PPI site [12]. Subsequent chemical modifications include the addition of aryl groups that are positioned between L68/E81 and are tethered by carbon linkers of differing length [1, 13, 14]. Notably, distinct conformations of the aryl rings were observed in the crystal structures of *Pa*‐Bfr KM‐5‐35 and KM‐5‐66 which have one and three carbon linkers respectively (Figure 6A,D). These compounds have been demonstrated to disrupt *Pa*‐Bfr:Bfd interactions and overall iron homeostasis in *A. baumannii* and *P. aeruginosa* .

**FIGURE 7:**
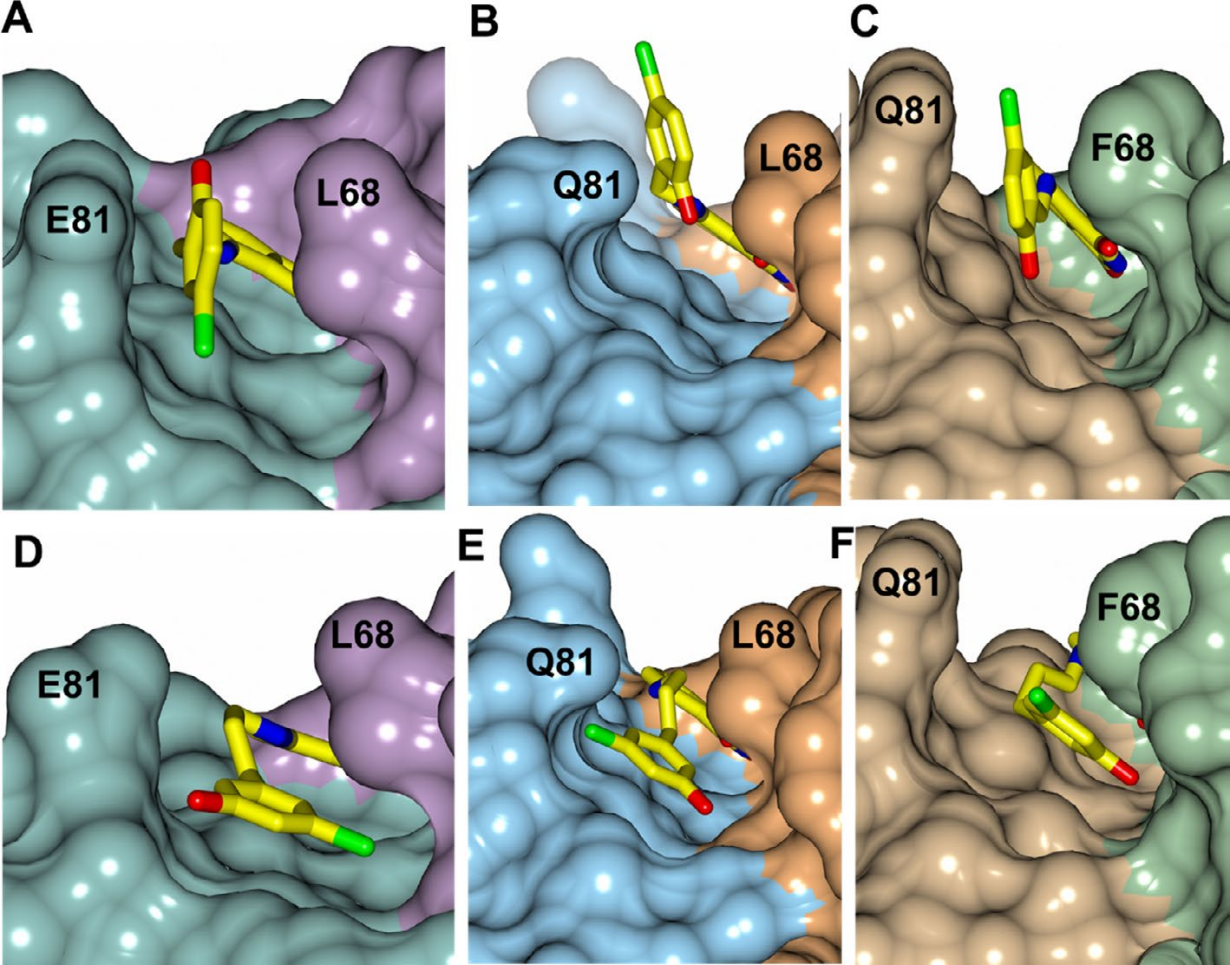
Surface representations showing the orientation of the aryl rings of KM‐5‐35 (A–C) and KM‐5‐66 (D–F) shown as yellow cylinders in the Bfr binding pocket. *Pa*‐Bfr, *Ab*‐Bfr and *Ba*‐Bfr are shown in panels (A, D), (B, E) and (C, F) respectively.

Given the similarity between aforementioned Bfr:Bfd models, docking experiments were performed using inhibitors KM‐5‐35 and KM‐5‐66 with the *Ab*‐Bfr and *Ba*‐Bfr crystal structures (Figure 6B,C,E,F). The phthalimide groups were restrained to hydrogen bond with the Bfr residues in the region spanning P69‐Q72. The top docked poses position the aryl groups between L68/Q81 (*Ab*) and F68/Q81 (*Ba*), similar to the experimental structures of the complexes formed by the compounds bound to *Pa*‐Bfr. The PPI sites in each Bfr structure contain a cleft where the “walls” are formed by the side chains of a glutamate (*Pa*‐Bfr) or glutamine (*Ab*‐Bfr and *Ba*‐Bfr) residue at position 81 and a leucine (*Pa*‐Bfr and *Ab*‐Bfr) or phenylalanine (*Ba*‐Bfr) (Figure 7). The poses for KM‐5‐35 position the aryl ring parallel to the wall of the PPI site (Figure 7A–C) whereas the aryl ring of KM‐5‐66 is rotated approximately 90° and nearly parallel to the floor (Figure 7D–F). The main difference is that aryl rings of the inhibitors in the docked models for *Ab* and *Ba* Bfr are rotated approximately 180° relative to the conformation observed for the aryl rings in the complexes with *Pa*‐Bfr. However, experimental structures of the inhibitors complexed to *Ab*‐Bfr and *Ba*‐Bfr are needed to confirm these details. It is of note that the aryl rings of the inhibitors have been shown to exhibit conformational disorder in various *Pa*‐Bfr inhibitor bound structures, often adopting two conformations. Given the similarity between these binding modes, it can be expected that KM‐5‐35, KM‐5‐66 and other similar inhibitors of the *Pa*‐Bfr:Bfd protein: protein complex will inhibit the *Ab* Bfr‐Bfd or *Ba*‐Bfr‐Bfd complex. In fact, a recent study has demonstrated that KM‐5‐35 and inhibitors of similar structure inhibit the *Ab* Bfr‐Bfd complex, dysregulate iron homeostasis, and kill *Acinetobacter baumannii* cells [14].

We previously reported the experimentally determined binding affinities (*K*_d_) of 9.0 μM (KM‐5‐35) [14] and 0.35 μM (KM‐5‐66) [13] to *Pa*‐Bfr. The binding affinity predictions of the docked ligands were computed using Boltz‐2 [38] which utilizes training based on biophysical affinity measurements such as *IC*_50_, *K*_d_ and *K*_i_ and outputs a general assessment of binding strength. This provides an approximation of binding affinity to a protein target, and we obtained the following results: *Ab*‐Bfr 17.5 μM (KM‐5‐35) and 4.0 μM (KM‐5‐66); *Ba*‐Bfr 10.5 μM (KM‐5‐35) and 7.4 μM (KM‐5‐66). Additionally, the computed affinities for *Pa*‐Bfr were 14.4 μM (KM‐5‐35) and 4.4 μM (KM‐5‐66) which differ by 1.6‐fold and 12.5‐fold respectively relative to the experimentally derived values. While these computational approaches can be useful to estimate ligand binding affinities, experimental techniques are needed to confirm binding strength of these ligands. In the context of this discussion, however, the computed *K*_d_ values for each of the inhibitors binding to *Pa*‐, *Ba*‐, or *Ab*‐Bfr are similar, confirming the observations that the Bfd binding site on Bfr is largely conserved and that inhibitors developed with the guidance of the *Pa*‐Bfr‐Bfd complex can be expected to also exert activity against *A. baumannii* and *B. abortus* cells.

## 4 Conclusion

Bacterioferritins are highly symmetric proteins that function in the maintenance of iron homeostasis in bacteria, protection from oxidative stress resulting from excess free iron, and provide iron to essential processes under iron limiting conditions. Bacterioferritins have been shown to be essential in pathogenesis and biofilm formation [3, 10]. The structure of Bfr from *Brucella abortus* shares a high degree of similarity to those obtained for *Pa*‐Bfr and *Ab*‐Bfr. Bfr proteins store insoluble Fe^3+^ as a mineralized complex with phosphate counter ions. Therefore, the sulfate and chloride ions observed in the 3‐fold and 4‐fold pores of each of the *Ba*‐Bfr structures suggest that its 3‐fold pores may also function in the transport of anions (presumably phosphate) across the protein coat, as has been shown for the 3‐fold pores in *Pa*‐Bfr. In this context, it is of note that the 4‐fold pores in *Ab*‐Bfr contain R148, instead of the most commonly observed N148. The presence of a positively charged side chain in the 4‐fold pores allowed for the unique observation of chloride ions in these pores. The ferroxidase center residues are identical in the *Pa*‐Bfr and *Ba*‐Bfr structures, suggesting similar modes of iron capture and oxidation.

Inspection of the Bfr binding sites on the structures of *Pa*‐Bfr, *Ab*‐Bfr, and *Ba*‐Bfr shows structural conservation. These observations, together with Alphafold models of *Ab*‐Bfd and *Ba*‐Bfd showing that these proteins exhibit structural conservation relative to the *Pa*‐Bfr structure, indicated that the complexes formed by cognate partners Bfr and Bfd in the three different bacteria may be very similar. Modeling experiments of the complexes strongly support this idea. Moreover, the experiments where inhibitors are docked to *Ab*‐Bfr and *Ba*‐Bfr show that the inhibitors bind these proteins with poses nearly identical to those observed in the structures of inhibitors bound to *Pa*‐Bfr. Recent work has demonstrated that these inhibitors can penetrate the *A. baumannii* cell, bind *Ab*‐Bfr and inhibit the *Ab* Bfr:Bfd complex formation, thus causing iron homeostasis dysregulation and bacterial cell death [14]. Consequently, it is reasonable to speculate that the same inhibitors will bind *Ba*‐Bfr and interfere with iron homeostasis in *Brucella abortus* . This could lead to new therapeutic approaches to brucellosis disease, which currently requires long treatments with multiple antibiotics and frequently fails [15].

## Author Contributions

**Lijun Liu:** methodology, writing – review and editing, formal analysis, software, investigation, validation. **Elizabeth K. Harmon:** writing – review and editing, formal analysis, investigation, resources, validation. **Justin K. Craig:** writing – review and editing, formal analysis, investigation, resources, validation. **Huili Yao:** formal analysis, investigation, writing – review and editing, methodology, validation. **Kevin P. Battaile:** writing – review and editing, formal analysis, investigation, methodology, software, data curation, resources, conceptualization. **David K. Johnson:** software, formal analysis, writing – review and editing, methodology, investigation, data curation, resources. **Sandhya Subramanian:** data curation, writing – review and editing, methodology, investigation. **Wesley C. Van Voorhis:** project administration, writing – review and editing, supervision, funding acquisition, conceptualization, methodology. **Mario Rivera:** conceptualization, funding acquisition, writing – original draft, writing – review and editing, methodology, formal analysis, project administration, supervision, resources. **Scott Lovell:** project administration, writing – original draft, funding acquisition, conceptualization, formal analysis, methodology, resources, writing – review and editing, supervision, visualization.

## Funding

This work was supported by the National Institute of Allergy and Infectious Diseases, 75N93022C00036, AI169344.

## Supporting information


**Data S1:** prot70109‐sup‐0001‐supinfo.pdf.

## Data Availability

The coordinates and structure factors for the *Ba*‐Bfr structures have been deposited to the Worldwide Protein Databank (wwPDB.org) with the accession codes 8SQP (*Ba*‐Bfr‐Apo1), 8SQQ (*Ba*‐Bfr‐Apo2), 8SQO (*Ba*‐Bfr‐Mg), 8SQR (*Ba*‐Bfr‐Fe1) and 8SQT (*Ba*‐Bfr‐Fe2). The expression clone is available for request online at https://www.ssgcid.org/available‐materials/expression‐clones/.
