# Supplementary material for: Structural Analysis and Inhibitor Modeling of Bacterioferritin From Brucella abortus
Source: Proteins. 2026 Jan 2;94(6):1170–81. doi: 10.1002/prot.70109 (PMC13136788; doi:10.1002/prot.70109)
Supplement: Supplementary file 1 — Data S1: prot70109‐sup‐0001‐supinfo.pdf. [file PROT-94-1170-s001.pdf]

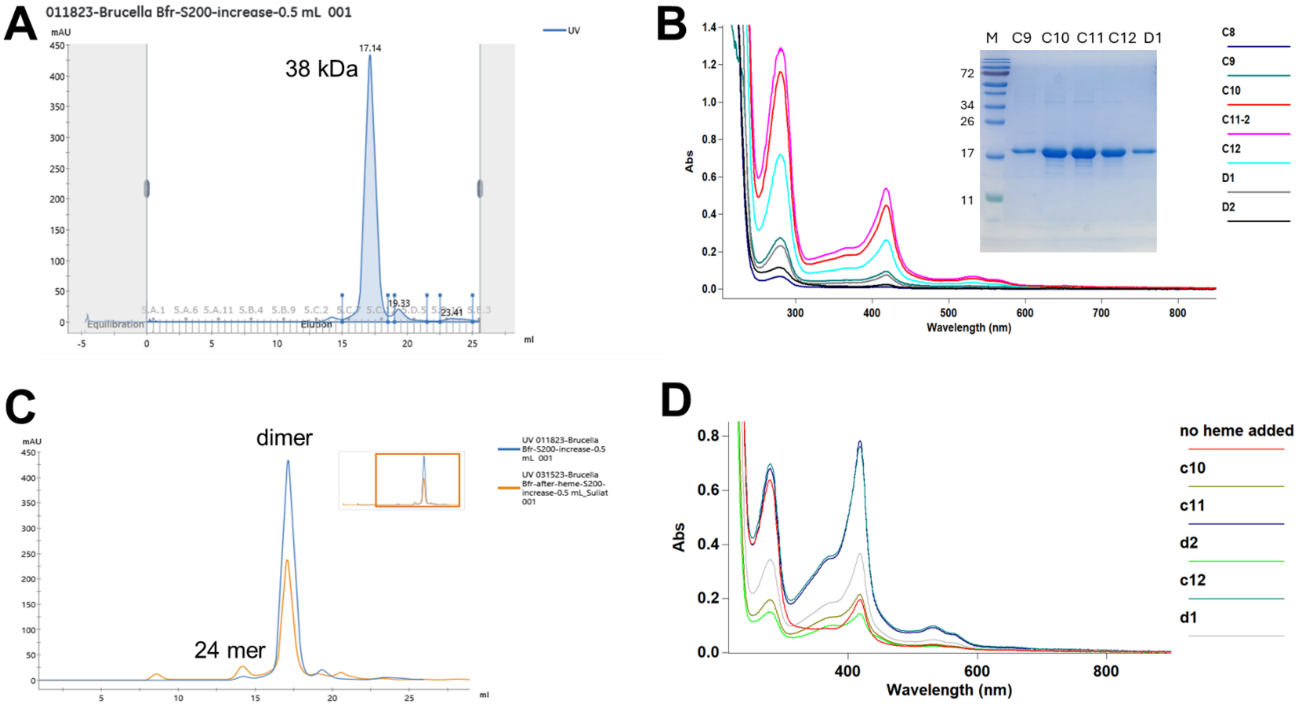

Supplemental Figure 1. Size exclusion chromatography of *Ba*-Bfr and UV-Vis spectra. **A**) Purification on a Superdex S200 increase column indicated that the protein is mostly dimeric. **B**) UV-Vis spectrum and SDS-PAGE of the Superdex 200 fractions showing incomplete heme incorporation based on the A420 nm/A280 nm ratio. **C**) Purification on a Superdex S200 increase column following titration with heme. The protein was mostly dimeric with a small amount of 24-mers observed. **D**) UV-Vis spectrum and SDS-PAGE of the Superdex 200 fractions in panel C showing a relative increase in the heme absorption at 420 nm indicating the incorporation of heme.

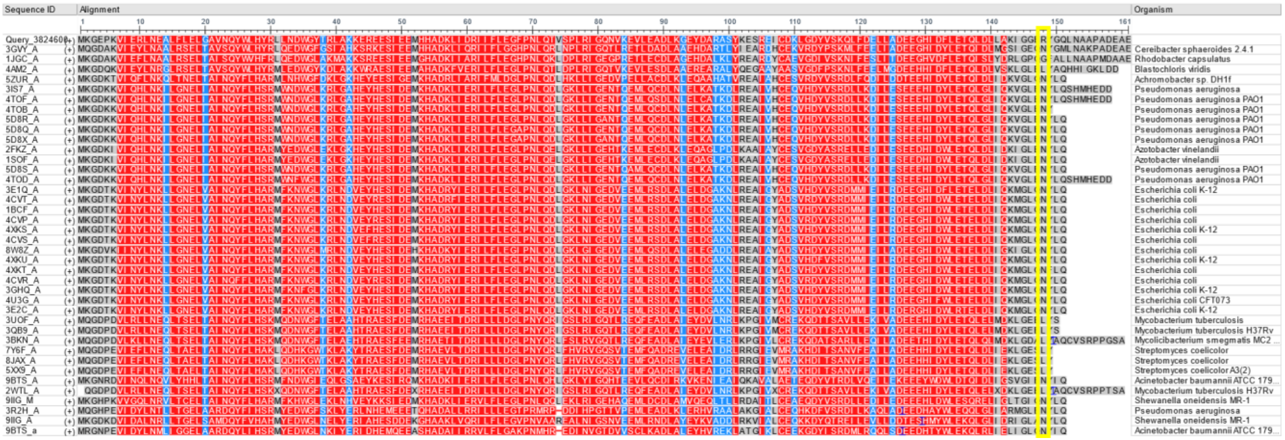

Supplemental Figure 2. Alignment of *Ba*-Bfr (Query_3824600) against other Bfr structures from the PDB. The homologous residue R148 in *Ba*-Bfr is highlighted yellow.

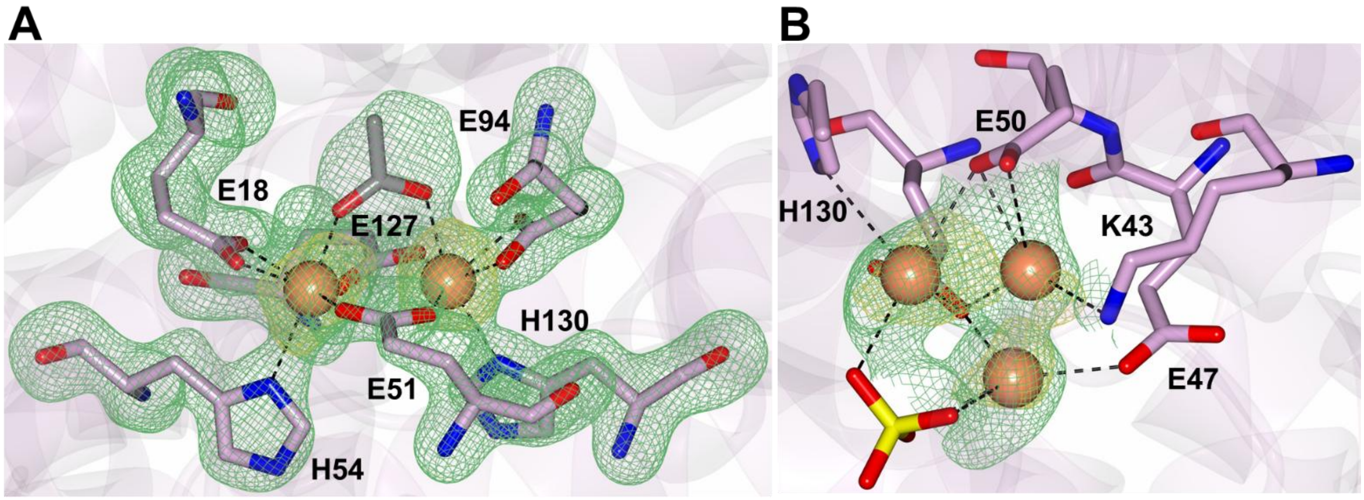

Supplemental Figure 3. Iron binding in *Ba*-Bfr. *Ba*-Bfr-Fe1 structure showing the Fo-Fc difference electron density (green mesh) and phased anomalous difference map (gold mesh) contoured at 3σ for the **A**) ferroxidase center and **B**) site 2 iron binding regions. The sulfate ion in stie 2 is drawn as yellow/red cylinders.

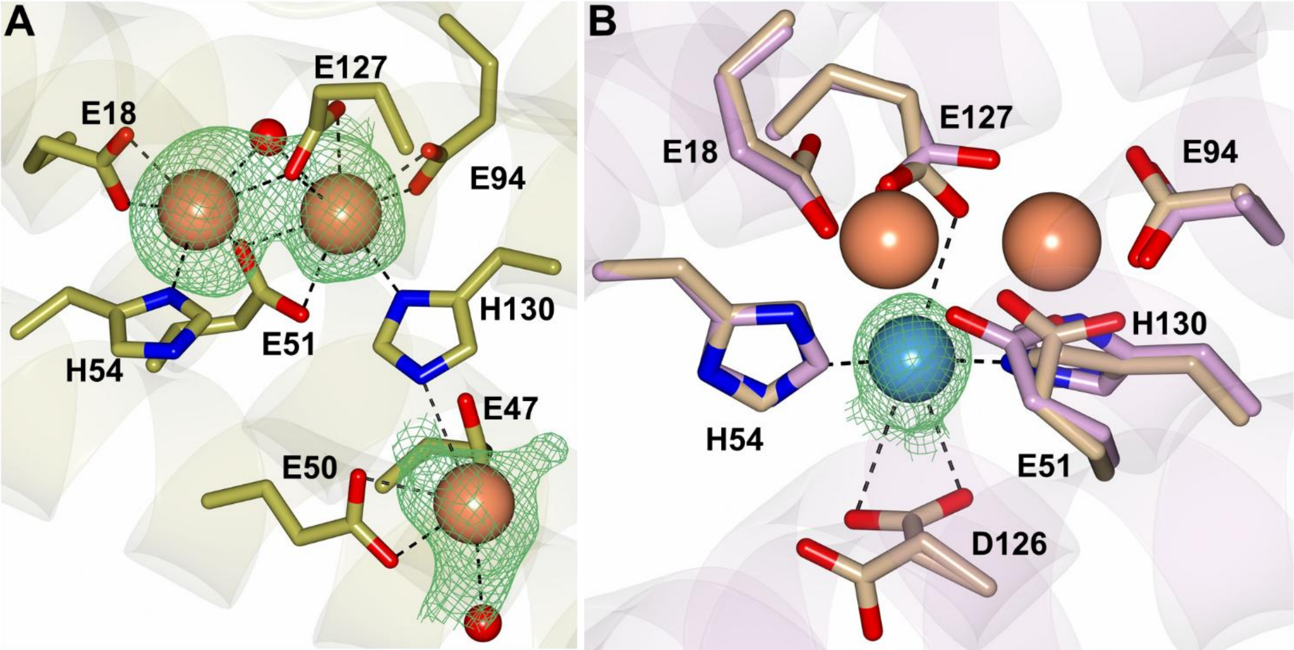

Supplemental Figure 4. Iron binding and magnesium binding in *Ba*-Bfr. **A**) *Ba*-Bfr-Fe2 structure showing the Fo-Fc difference electron density (green mesh) contoured at 3σ for the iron ions. **B**) Ba-Bfr-Mg (tan) showing the magnesium ion (blue sphere) relative to the Ba-Bfr-Fe1 structure (plum). Iron ions are drawn as coral spheres in each panel.

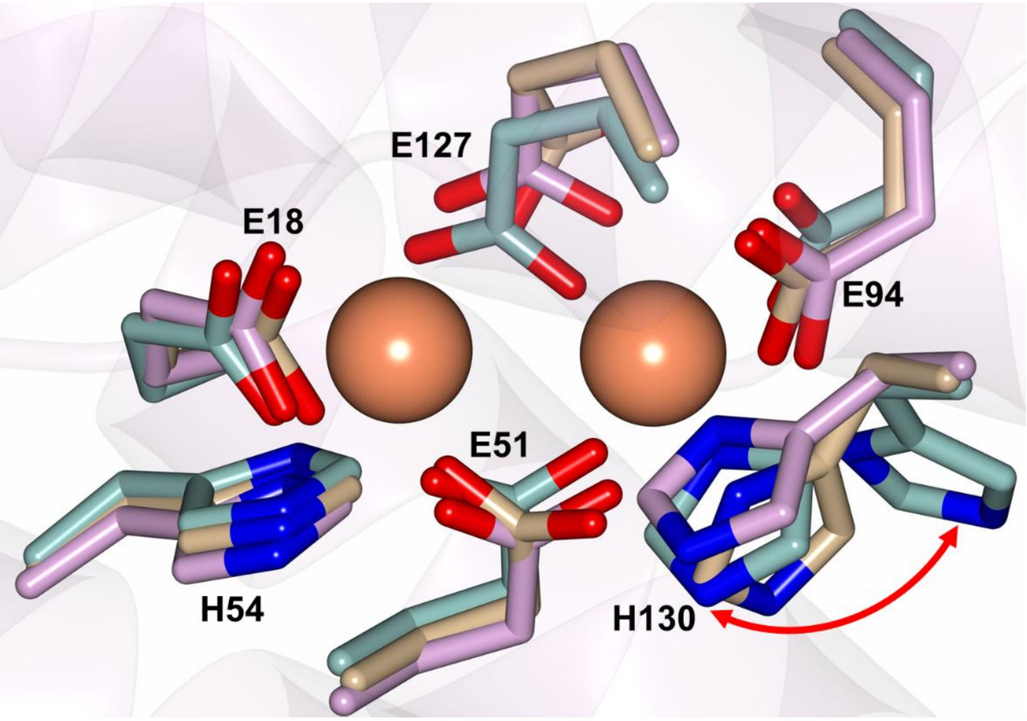

Supplemental Figure 5. Superposition of *Ba*-Bfr-Fe1 (plum) with apo (tan, 3IS7) and iron bound (teal, 3IS8) *Pa*-Bfr showing the positions of the ferroxidase center residues. Iron bound *Pa*-Bfr has two distinct conformations at H130 as indicated by the red arrow.
